# Supplementary material for: A new validated Lymphoedema-specific Patient Reported Outcome Measure (LYMPROM) for adults with Lymphoedema
Source: PLoS One. 2025 May 23;20(5):e0315314. doi: 10.1371/journal.pone.0315314 (PMC12101676; doi:10.1371/journal.pone.0315314)
Supplement: Table S1 — Correlation matrix for LYMPROM© items. (DOCX) [file pone.0315314.s001.docx]

# Supplementary Table S1

Correlation matrix for LYMPROM© items.

| Item | Pain | Heaviness | Home Life | Personal Care | Work | Finances | Body Image | Intimacy /desirability | Walking | Anxiety | Hobbies | Holidays | Shopping |
| --- | --- | --- | --- | --- | --- | --- | --- | --- | --- | --- | --- | --- | --- |
| Pain | - |  |  |  |  |  |  |  |  |  |  |  |  |
| Heaviness | 0.477 (0.401, 0.547) [432] | - |  |  |  |  |  |  |  |  |  |  |  |
| Home Life | 0.598 (0.533, 0.655) [432] | 0.623 (0.561, 0.677) [431] | - |  |  |  |  |  |  |  |  |  |  |
| Personal Carer | 0.536 (0.466, 0.600) [431] | 0.531 ().459, 0.595) [430] | 0.747 (0.702, 0.786) (430) | - |  |  |  |  |  |  |  |  |  |
| Work | 0.543 (0.338, 0.699) [61] | 0.665 (0.497, 0.786) [61] | 0.673 (0.507, 0.791) [61] | 0.430 (0.197, 0.616) [60] | - |  |  |  |  |  |  |  |  |
| Finances | 0.648 (0.460, 0.780) [54] | 0.606 (0.403, 0.752) [54] | 0.773 (0.637, 0.862) [54] | 0.609 (0.408, 0.754) [54] | 0.571 (0.334, 0.741) [45] | - |  |  |  |  |  |  |  |
| Body Image | 0.399 (0.317, 0.479) [428] | 0.420 (0.339, 0.495) [427] | 0.490 (0.414, 0.559) [427] | 0.462 (0.383, 0.533) [426] | 0.619 (0.435, 0.753) [61] | 0.421 (0.170, 0.621) [53] | - |  |  |  |  |  |  |
| Intimacy / desirability | 0.471 (0.308, 0.606) [107] | 0.464 (0.299, 0.601) [106] | 0.521 (0.368, 0.647) [107] | 0.458 (0.293, 0.597) [106] | 0.604 (0.378, 0.763) [45] | 0.589 (0.354, 0.754) [44] | 0.827 (0.755, 0.880) [105] | - |  |  |  |  |  |
| Walking | 0.476 (0.400, 0.546) [432] | 0.625 (0.564, 0.679) [431] | 0.708 (0.658, 0.752) [431] | 0.639 (0.580, 0.692) [431] | 0.580 (0.382, 0.727) [60] | 0.705 (0.539, 0.818) [54] | 0.431 (0.350, 0.505) [427] | 0.474 (0.312, 0.610) [106] | - |  |  |  |  |
| Anxiety | 0.488 (0.413, 0.557) [431] | 0.436 (0.356, 0.509) [430] | 0.572 (0.505, 0.633) [430] | 0.533 (0.462, 0.598) [429] | 0.582 (0.387, 0.728) [61] | 0.594 (0.388, 0.743) [54] | 0.658 (0.600, 0.709) [426] | 0.709 (0.600, 0.792) [107] | 0.491 (0.416, 0.560) [430] | - |  |  |  |
| Hobbies | 0.461 (0.383, 0.532) [429] | 0.441 (0.362, 0.515) [428] | 0.556 (0.487, 0.619) [428] | 0.524 (0.451, 0.589) [428] | 0.598 (0.403, 0.741) [59] | 0.617 (0.416, 0.760) [53] | 0.623 (0.561, 0.678) [424] | 0.657 (0.532, 0.753) [105] | 0.553 (0.484, 0.616) [429] | 0.657 (0.600, 0.708) [427] | - |  |  |
| Holidays | 0.376 (0.225, 0.508) [143] | 0.486 (0.349, 0.602) [143] | 0.572 (0.449, 0.673) [142] | 0.502 (0.367, 0.616) [141] | 0.698 (0.509, 0.823) [45] | 0.684 (0.479, 0.818) [42] | 0.709 (0.616, 0.782) [142] | 0.737 (0.613, 0.825) [76] | 0.566 90.443, 0.669) [142] | 0.737 (0.651, 0.804) [142] | 0.759 (0.679, 0.822) [140] | - |  |
| Shopping | 0.417 (0.336, 0.492) [433] | 0.502 (0.428, 0.570) [432] | 0.432 (0.446, 0.584) [432] | 0.487 (0.412, 0.556) [431] | 0.532 (0.323, 0.691) [61] | 0.420 (0.172, 0.618) [54] | 0.581 (0.515, 0.641) [428] | 0.645 (0.519, 0.744) [107] | 0.577 (0.510, 0.637) [432] | 0.547 (0.477, 0.610) [431] | 0.510 (0.437, 0.577) [429] | 0.651 (0.545, 0.736) [143] | - |

Items are in original LYMPROM© order; cells contain: the Pearson correlation, (lower 95% CI, upper 95% CI), [n]
