# Supplementary material for: A new validated Lymphoedema-specific Patient Reported Outcome Measure (LYMPROM) for adults with Lymphoedema
Source: PLoS One. 2025 May 23;20(5):e0315314. doi: 10.1371/journal.pone.0315314 (PMC12101676; doi:10.1371/journal.pone.0315314)
Supplement: Table S2 — Correlation matrix for LYMPROM© items (coded). (DOCX) [file pone.0315314.s002.docx]

# Supplementary Table S2

Correlation matrix for LYMPROM© items (coded).

| Item | PH1 | PH2 | PH3 | SH1 | SH2 | SH3 | SH4 | SH5 | SH6 | SH7 | EH1 | EH2 | EH3 |
| --- | --- | --- | --- | --- | --- | --- | --- | --- | --- | --- | --- | --- | --- |
| PH1 | - |  |  |  |  |  |  |  |  |  |  |  |  |
| PH2 | 0.477 | - |  |  |  |  |  |  |  |  |  |  |  |
| PH3 | 0.476 | 0.625 | - |  |  |  |  |  |  |  |  |  |  |
| SH1 | 0.598 | 0.623 | 0.708 | - |  |  |  |  |  |  |  |  |  |
| SH2 | 0.536 | 0.531 | 0.639 | 0.747 | - |  |  |  |  |  |  |  |  |
| SH3 | 0.543 | 0.665 | 0.580 | 0.673 | 0.430 | - |  |  |  |  |  |  |  |
| SH4 | 0.648 | 0.606 | 0.705 | 0.773 | 0.609 | 0.571 | - |  |  |  |  |  |  |
| SH5 | 0.461 | 0.441 | 0.553 | 0.556 | 0.524 | 0.598 | 0.617 | - |  |  |  |  |  |
| SH6 | 0.376 | 0.486 | 0.566 | 0.572 | 0.502 | 0.698 | 0.684 | 0.759 | - |  |  |  |  |
| SH7 | 0.417 | 0.502 | 0.577 | 0.432 | 0.487 | 0.532 | 0.420 | 0.510 | 0.651 | - |  |  |  |
| EH1 | 0.399 | 0.420 | 0.431 | 0.490 | 0.462 | 0.619 | 0.421 | 0.623 | 0.709 | 0.581 | - |  |  |
| EH2 | 0.471 | 0.464 | 0.474 | 0.521 | 0.458 | 0.604 | 0.589 | 0.657 | 0.737 | 0.645 | 0.827 | - |  |
| EH3 | 0.488 | 0.436 | 0.491 | 0.572 | 0.533 | 0.582 | 0.594 | 0.657 | 0.737 | 0.547 | 0.658 | 0.709 | - |

Items coded using LYMPROM© domains: Physical health (PH1-PH3), Social health (SH1-SH7), Emotional health (EH1-EH3).
